# Effects of Two Doses of Organic Extract-Based Biostimulant on Greenhouse Lettuce Grown Under Increasing NaCl Concentrations

**DOI:** 10.3389/fpls.2018.01870

**Published:** 2019-01-07

**Authors:** Roberta Bulgari, Alice Trivellini, Antonio Ferrante

**Affiliations:** ^1^Department of Agricultural and Environmental Sciences – Production, Landscape, Agroenergy, University of Milan, Milan, Italy; ^2^Institute of Life Sciences, Scuola Superiore Sant’Anna Pisa, Pisa, Italy

**Keywords:** biostimulant, *Lactuca sativa* L., salinity, abiotic stress, non-destructive measurements, biochemical analyses

## Abstract

The enhancement of plant tolerance toward abiotic stresses is increasingly being supported by the application of biostimulants. Salinity represents a serious problem in the Mediterranean region. To verify the effects deriving from the application of biostimulants, trials on Romaine lettuce plants under salt exposure were performed, in greenhouse. Plants were subjected to three NaCl solutions with 0.8, 1.3, and 1.8 dS/m of electrical conductivity. The volume of the solution was 200 mL/plant and delivered every 3 days. Biostimulant treatments started after crop establishment and were: control (water) and two doses (0.1 or 0.2 mL/plant) of the commercial biostimulant Retrosal^®^ (Valagro S.p.A), containing calcium, zinc, and specific active ingredients. Four Retrosal^®^ treatments were applied, every 7 days, directly to the substrate. Non-destructive analyses were conducted to assess the effects on leaf photosynthetic efficiency. At harvest, plants fresh weight (FW) and dry weight were determined, as well as the concentration of chlorophylls, carotenoids, total sugars, nitrate, proline, and abscisic acid (ABA). The biostimulant tested increased significantly the FW of lettuce (+65% in the highest dose) compared to controls. Results indicate that treatments positively affected the chlorophyll content measured *in vivo* (+45% in the highest dose) and that a general positive effect was observable on net photosynthesis rate. Retrosal^®^ seems to improve the gas exchanges under our experimental conditions. The total sugars levels were not affected by treatments. Biostimulant allowed maintaining nitrate concentration similar to the untreated and unstressed controls. The increasing levels of water salinity caused a raise in proline concentration in control plants (+85%); biostimulant treatments at 0.2 mL/plant dose kept lower the proline levels. All plants treated with the biostimulant showed lower value of ABA (-34%) compared to controls. Results revealed that Retrosal^®^ is able to stimulate plant growth independently from the salinity exposure. However, treated plants reached faster the commercial maturity stage. The fresh biomass of control at the end of experiment, after 30 days, ranged from 15 to 42 g/head, while in biostimulant treated plants ranged from 45 to 94 g/head. The product applied at maximum dose seems to be the most effective in our experimental conditions.

## Introduction

Abiotic stresses are among the primary causes of crop losses worldwide, reducing average yields for most major crops by more than 50% (Bray et al., 2000; La Pena and Hughes, 2007). The reduction of yield under abiotic stress is mainly due to the energy that crops have to use for adaptation. These yield losses are usually known as “fitness cost” of the crops.

Several approaches have been employed to enhance abiotic stress tolerance. However, some of them are considered time-consuming. For instance, conventional breeding requires laborious selection and the process of several generations of crossing, selfing, and testing plants for tolerance (Ashkani et al., 2015). On the other hand, the new plant breeding techniques allow to develop new tolerance traits within a given species through genetic engineering in a short period of time, but they are currently forbidden in many countries (Savvides et al., 2016). An interesting and sustainable alternative can be provided by the application of biostimulants that strengthen the plants to more successfully tolerate future abiotic stress conditions. Biostimulants have been promoted for their ability to counteract abiotic stresses in plants and their mode of action is increasingly studied. These products are able to counteract environmental stress such as water deficit, soil salinization, and exposure to sub-optimal growth temperatures (du Jardin, 2015; Pokluda et al., 2016; Rouphael et al., 2017a; Van Oosten et al., 2017; Desoky et al., 2018; Di Stasio et al., 2018; Masondo et al., 2018; Ugena et al., 2018). Salinity, in particular, is considered one of the main environmental factor that affects plant growth and metabolism in many Mediterranean areas, leading to severe damage, turgor loss and severe inhibition of growth (Borgognone et al., 2014; Lucini et al., 2015; Taïbi et al., 2016; Rouphael et al., 2017b). It represents a serious problem for commercial horticulture with substantial loss of productivity (Xu and Mou, 2016; Orsini et al., 2018), especially in the Mediterranean region where the electric conductivity of water is often higher and overcome the crop threshold sensitivity (Colla et al., 2010). Sodium chloride (NaCl) is the main salt presents in saline environments along the seaside production areas (Viégas et al., 2001). Salinity stress can be induced by the salts accumulated in the soils that are distributed through the irrigation water. In the case of cultivations in open field along the coast, plants can also suffer from aerosol marine due to salt accumulation on the leaves. Plants exposure to salinity results in stunted growth, nutrient imbalance, and reduction in water potential (Munns and Termaat, 1986; Blaylock, 1994; Marschner, 1995; Maas and Grattan, 1999; Shaheen et al., 2013). Salt stress in plants induces similar effects of drought (Munns, 2002; Chaves et al., 2009); in fact, osmotic stress can be a consequence of either salt or drought (Forni et al., 2017). Plants have different degree of tolerance that depends from various adaptation methods and metabolic plasticity. Salt stress could also alter several metabolic processes in plants, such as photosynthesis (Agastian et al., 2000; Sayyad-Amin et al., 2016), respiration (Moud and Maghsoudi, 2008), phytohormone regulation, protein synthesis, nitrogen assimilation, and can also generate secondary oxidative stress (Flowers, 2004; Van Breusegem and Dat, 2006; Colla et al., 2010). Salinity stress induces a wide activation of the biosynthesis of bioactive compounds, which are able to reduce cell damage (Cavaiuolo et al., 2015). Several transcription factors have been identified and found differently expressed in stressed leaves. To reduce the interferences with cell physiology, salts are accumulated in vacuoles and in older leaves. Plants defense mechanisms are oriented to reduce the water uptake to avoid salts loading in the cells. Physiological alterations to enhance plant tolerance to salt stress involve the plant hormone abscisic acid (ABA) (Ferrante et al., 2011; Trivellini et al., 2016). For example, salt-induced ABA accumulation was reported to activate ABA-dependent signaling pathways (Zhu, 2002), which in turn led to adaptation.

To verify the effects deriving from the applications of biostimulants, trials on lettuce plants under salinity exposure were performed. Lettuce is in fact considered to be a moderately salt sensitive crop (Shannon and Grieve, 1998; Fernandez et al., 2016) and it is one of the most important leafy vegetable cultivated in the Mediterranean area, where saline water is frequently used for irrigation. The salinity threshold for this vegetable species is in average 1.3 dS/m (Ayers et al., 1951; Maas and Hoffman, 1977; Shannon and Grieve, 1998; Andriolo et al., 2005; Ünlükara et al., 2010), as observed in the majority of the cultivars or varieties. The effect of biostimulants can be ascribed to the improvement of the osmotic adjustment in cells by the accumulation of osmotic metabolites and the sequestration of salts in vacuoles, interfering with other compounds. The hypothesis of this work was to evaluate if an organic extract-based biostimulant, containing calcium, zinc, and specific active ingredients could enhance the tolerance against salinity in lettuce, since bioactive compounds and calcium can improve plant response and adaptation.

## Materials and Methods

### Plant Material and Treatments

Romaine lettuce (*Lactuca sativa* ‘Longifolia’) plantlets were obtained from a local nursery. Two-week-old plantlets were transplanted in 22 cm diameter plastic pots (eight pots/treatment), on a peaty substrate, in a glasshouse of the Faculty of Agricultural and Food Sciences of Milan, under controlled conditions. The environmental conditions during the experimental period were 22–33°C, with a relative of humidity ranging from 70–80%, and 600–800 μmol m^-2^ s^-1^. Nutrients were directly added to the substrate and were supplied by providing 14 g of slow-release fertilizer containing (NPK+MgO +SO_3_: 14-7-17 + 2 +20). The first application, 7 g, was performed at transplanting, mixing the fertilizer with peat, and the second one (7 g) was carried out during cultivation. The density was 10 plants/m^2^. Three NaCl solutions, with increasing concentration [0.8, 1.3, and 1.8 dS/m of electrical conductivity (EC)] were prepared in laboratory. These EC levels were obtained by adding 0.5 g L^-1^ (1.3 dS/m) or 0.8 g L^-1^ (1.8 dS/m) NaCl; the 0.8 dS/m was maintained using only tap water. The first saline solution can be considered not stressful for lettuce, the second one as a threshold of salinity tolerance, while the last one as stressful for the crop considered. The volume of the saline solution was 200 mL/plant and delivered every 3 days. EC values of the substrate at harvest are reported in Supplementary Table S2. Treatments conditions were: control (water) and the commercial biostimulant Retrosal^®^ (Valagro S.p.A) applied every 7 days at 10 or 20 L/ha dose, which correspond to 0.1 or 0.2 mL plant^-1^. The biostimulant Retrosal^®^ is an organic mix with high concentration of carboxylic acids, containing calcium oxide (CaO) 8.0% (w/w) soluble in water and 1.4% complexed by ammonium ligninsulfonate, Zinc (Zn) 0.2% (w/w) soluble in water and 0.2% (w/w) chelated by EDTA. Calcium complexed by ammonium ligninsulfonate is stable in the pH comprised from 3 to 9, while the Zn chelated with EDTA is stable in pH comprised from 4 to 11.

The irrigation was carried out considering the substrate moisture content and the amount of water was determined to maintain the 80% of substrate water availability.

Lettuce plants were harvested when the first treatment reached the commercial maturity stage, after 30 days of cultivation. Fresh weight (FW), dry biomass, and dry matter were determined weighting the whole lettuce head before and after an over-dry period (4 days) at 75°C in a ventilated over. At harvest, non-destructive analyses were conducted on leaves and then fresh leaf tissues were immediately stored at -80 or -20°C until use for biochemical analyses.

### Destructive Analyses

#### Chlorophylls and Carotenoids

Chlorophyll *a+b* and total carotenoids concentrations were determined spectrophotometrically at harvest. Leaf tissue (30–50 mg) was extracted using 100% (v/v) methanol, for 24 h at 4°C in the dark; afterward quantitative determination of pigments was carried out. Absorbance readings were measured at 665.2 and 652.4 nm for chlorophylls and 470 nm for total carotenoids. Pigment levels were calculated by Lichtenthaler’s (1987) formula and expressed on the basis of tissue FW.

#### Total Sugars

Leaf tissue (1 g) was homogenized in 3 mL of distilled water and centrifuged at 3000 ×*g* (ALC centrifuge-model PK130R) for 15 min at room temperature (RT). Total sugars were assayed according to the anthrone assay. About 1 g of leaf tissue was homogenized in 3 mL of distilled water and centrifuged at 3000 ×*g*, for 15 min, at RT. Anthrone (0.2 g) was melted in 100 mL of H_2_SO_4_ and shook for 30–40 min. 1 mL of the leaf tissue extract was added to 5 mL of anthrone solution, cooled in ice for 5 min and mixed thoroughly. Samples were incubated at 95°C for 5 min and then cooled on ice (Yemm and Willis, 1954). Absorbance was read at 620 nm and the levels were calculated referring to glucose calibration curve (Cocetta et al., 2015).

#### Leaf Nitrate Concentration

Nitrate concentration was measured by the salicylsulfuric acid method (Cataldo et al., 1975). One gram of fresh leaf tissue was homogenized (mortar and pestle) in 3 mL of distilled water. The extract was centrifuged at 3000 ×*g* for 15 min at RT and the recovered supernatant was used for the colorimetric determination. Twenty μL of sample were added to 80 μL of 5% (w/v) salicylic acid dissolved in H_2_SO_4_ plus 3 mL of 1.5N NaOH. Samples were cooled at RT and absorbance at 410 nm was measured. Nitrate concentration was calculated referring to a KNO_3_ standard calibration curve.

#### Proline

Proline was determined with a colorimetric assay, as described by Abrahám et al. (2010). Lettuce leaves (0.5 g) were ground in 10 mL of sulfosalicylic acid (3%). Tubes were kept on ice and, subsequently, samples were centrifuged for 5 min, at RT, at 3800 ×*g* for 10 min. In a separate tube was prepared the reaction mixture: 100 μL of 3% sulfosalicylic acid, 200 μL of glacial acetic acid, 200 μL of acidic ninhydrin. Then 100 μL from the supernatant of the plant extract were added and the tubes were mixed well. Tubes were incubated at 96°C for 60 min. Then samples were put in ice. Afterward, 1 mL of toluene was added to the reaction mixture and samples were vortexed for 20 s. Tubes were left on the bench for 5 min to allow the separation of the organic and water phases. The chromophore containing toluene was removed into a fresh tube. Absorbance readings were performed at 520 nm using toluene as reference. Proline concentration was determined using a standard concentration curve and calculated based on the FW.

#### Abscisic Acid

Abscisic acid was determined by an indirect enzyme linked immuno-sorbent assay (ELISA) based on the use of DBPA1 monoclonal antibody, raised against S(+)-ABA (Vernieri et al., 1989). About 1 g of lettuce leaf was homogenized (mortar and pestle) in 3 mL of distilled water. The extract was centrifuged at 3000 ×*g* for 15 min at RT and the recovered supernatant was used for the analysis. The ELISA was performed as described by Trivellini et al. (2016).

#### Mineral Element Determinations

About 300 mg dry weight (DW) was mineralized at 120°C in 5 mL 14.4 M HNO_3_, clarified with 1.5 mL 33% H_2_O_2_ and finally dried at 80°C. The mineralized material was solubilized in 5 mL 1 M HNO_3_ and filtered on a 0.45-μm nylon membrane. Mineral content (Na, Ca, Mg, K, Mn, Fe, Cu, Zn, Cd, and P) was measured by inductively-coupled plasma techniques (ICP-MS; Varian 820-MS, ICP Mass Spectrometer).

### Non-destructive Measurements

#### Leaf Gas Exchange

Leaf gas exchange rates were measured using the portable infrared gas exchange system CIRAS-1 (PP Systems, Hitchin, United Kingdom), operated in open-configuration with controlled temperature, CO_2_ concentration, and vapor pressure. Measurements were carried out on a fully expanded leaf between 09:00 and 13:00 h IT time. During the recording time, the light intensity in the cuvette was fixed to 1000 μmol m^-2^ s^-1^ and CO_2_ concentration was set to 350 ppm.

#### Chlorophyll *a* Fluorescence

Chlorophyll *a* fluorescence was measured using a hand-portable fluorometer (Handy PEA, Hansatech, King’s Lynn, United Kingdom). Leaves were dark-adapted for 30 min. Using a leaf clip (4 mm diameter), a rapid pulse of high-intensity light of 3000 μmol m^-2^ s^-1^ (600 W m^-2^) was administered to the leaf inducing fluorescence. The fluorescence parameters were calculated automatically by the used device. Modulated chlorophyll *a* fluorescence was also determined, to measure the fluorescence yield even in full sunlight, using a field portable pulse modulated chlorophyll fluorometer (FMS2_,_ Hansatech, King’s Lynn, United Kingdom).

#### Chlorophyll Measurements *in vivo*

Chlorophyll content was estimated *in vivo* with a chlorophyll meter (CL-01, Hansatech, United Kingdom). This device provides an indication of green color of leaves and it determines relative chlorophyll content using dual wavelength optical absorbance (620 and 940 nm wavelength).

### Statistical Analysis

Statistical analysis was performed with GraphPad Prism 6. All data were compared by using two-way ANOVA, with Tukey’s multiple comparison test. Where the interaction between the two factors BS treatments and EC levels (AxB) was significant, data were subjected to one-way ANOVA, comparing all treatments each other. On the contrary, where AxB interaction was not significant, the effect of BS treatments and EC levels was separately evaluated, comparing the respective means.

Each treatment was composed by eight plants randomly distributed on the greenhouse bench. The non-destructive analyses were performed on four biological replications, while destructive analyses on three biological replications. Additional information is reported in each figure legends.

## Results

### Fresh Yield, Dry Biomass Production, and Percentage Dry Matter of Plants

**FIGURE 1:**
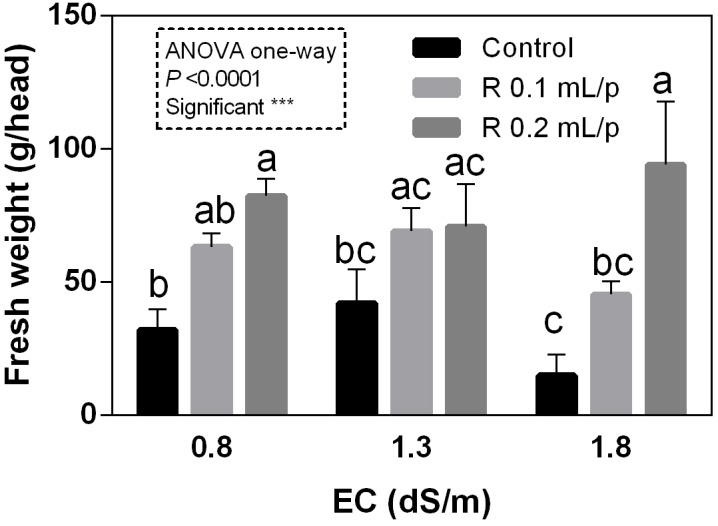
Fresh weight (FW) (g/head) of Romaine lettuce plants, at harvest, subjected to different levels of salinity (0.8, 1.3, and 1.8 dS/m) and treated with water (control) or Retrosal^®^ at 0.1 or 0.2 mL/plant dose. Values are means ± SE (*n* = 3). Data were subjected to two way ANOVA and Tukey’s multiple comparison test was used for evaluating the differences among means at (^∗^*p* < 0.05, ^∗∗∗^*p* < 0.001). Since interaction was significant, data were also subjected to one-way ANOVA. Different letters indicate statistical differences for *p* < 0.05.

**FIGURE 2:**
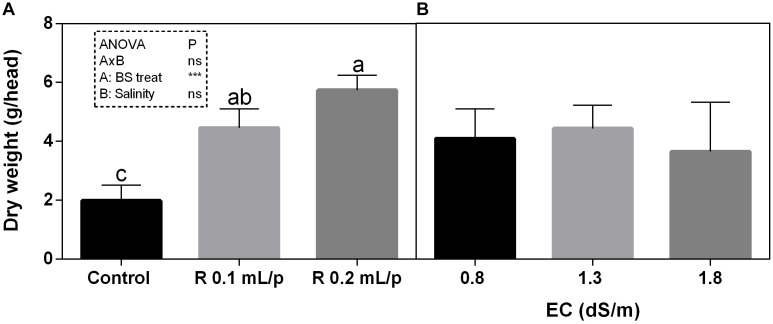
Dry weight (DW) (g/head) of Romaine lettuce plants, at harvest, subjected to different levels of salinity (0.8, 1.3, and 1.8 dS/m) and treated with water (control) or Retrosal^®^ at 0.1 or 0.2 mL/plant dose. Data were subjected to two way ANOVA and Tukey’s multiple comparison test was used for evaluating the differences among means at (^∗^*p* < 0.05, ^∗∗∗^*p* < 0.001). Since there was not significant AxB interaction, the effect of BS treatments **(A)** and EC levels **(B)** was evaluated separately, comparing the respective means ± SE (*n* = 3). Different letters indicate statistical differences for *p* < 0.05.

The FW of the whole lettuce plants (g/head) was determined at harvest, when the first treatment reached the commercial development stage (about 80 g/head). Statistical analysis showed that the interaction between salinity and biostimulant treatments was significant for *p* < 0.05. The biostimulant factor had a significant effect for *p* < 0.0001. Therefore, all treatments were analyzed using one-way ANOVA (Figure 1). Biostimulant significantly increased the FW of treated lettuce plants compared to control in all EC levels. The application of Retrosal^®^ at 0.2 mL/plant dose increased more than double this parameter, also under salinity (Figure 1). The DW of plants was statistically different among the biostimulant treatments for *p* < 0.001 (Figure 2). Statistical differences were found at 0.8 and 1.8 dS/m EC. The highest DW value was found in plants treated with 0.2 mL/plant dose in 1.8 dS/m salinity level. No significant difference was observed for the dry matter percentage that in average was 7% (Supplementary Figure S1).

#### Leaf Gas Exchange Measurements

**FIGURE 3:**
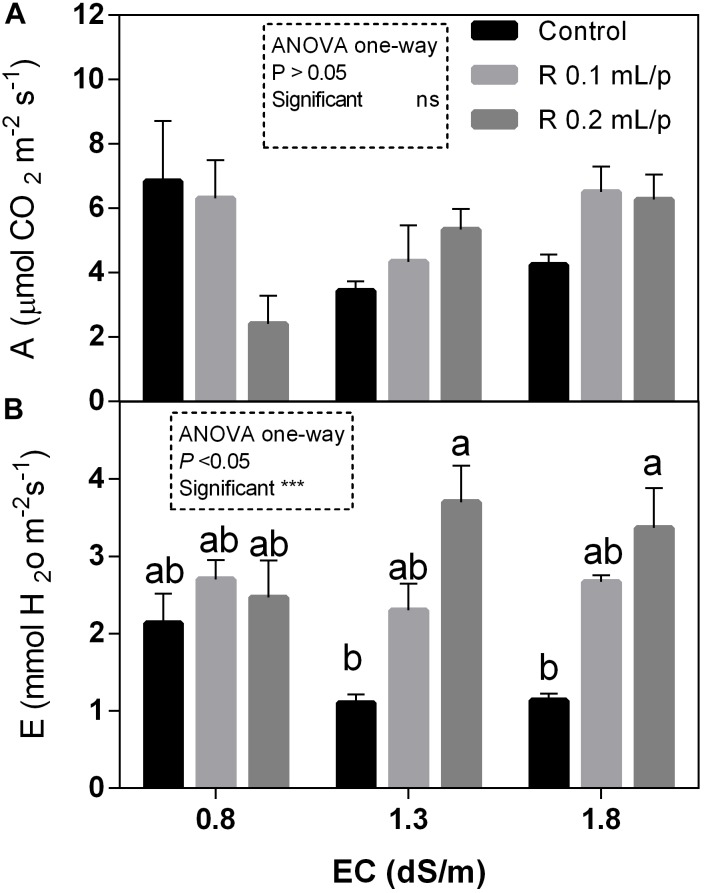
Leaf gas exchanges parameters [net photosynthesis **(A)** and transpiration **(B)**] measured *in vivo* in Romaine lettuce plants, at harvest. Plants were subjected to different levels of salinity (0.8, 1.3, and 1.8 dS/m) and treated with water (control) or Retrosal^®^ at 0.1 or 0.2 mL/plant dose. Values are means ± SE (*n* = 4). Data were compared by using two way ANOVA, with Tukey’s multiple comparison test (^∗^*p* < 0.05, ^∗∗∗^*p* < 0.001). Since interaction was significant, data were also subjected to one-way ANOVA. Different letters indicate statistical differences for *p* < 0.05.

**FIGURE 4:**
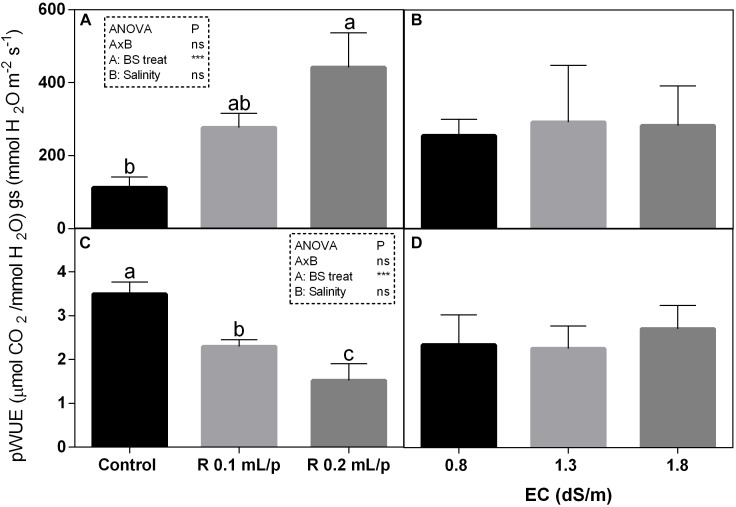
Leaf gas exchanges parameters [stomatal conductance **(A,B)** and photosynthetic water use efficiency (pWUE) **(C,D)**] measured *in vivo* in Romaine lettuce plants, at harvest. Plants were subjected to different levels of salinity (0.8, 1.3, and 1.8 dS/m) and treated with water (control) or Retrosal^®^ at 0.1 or 0.2 mL/plant dose. Data were compared by using two way ANOVA, with Tukey’s multiple comparison test (^∗^*p* < 0.05, ^∗∗∗^*p* < 0.001). Since there was not significant AxB interaction, the effect of BS treatments and EC levels was evaluated separately, comparing the respective means ± SE (*n* = 3). Different letters indicate statistical differences for *p* < 0.05.

The statistical analysis showed that net photosynthesis data (A) had significant interaction (*p* < 0.05) between biostimulant and salinity while no significant differences were observed among biostimulant or salinity treatments. Subsequently, data were also analyzed using one-way ANOVA but no significant differences were found (Figure 3A). The stomatal conductance (Figures 4A,B), that indicates the degree of exchange of CO_2_ and water vapor between environment and inner leaf, showed low values in control plants, mainly under salinity. Statistical analysis showed that there was not significant interaction between factors and in the salinity. Significant differences were observed for the biostimulant (*p* < 0.001). Stomatal conductance showed significant differences in plants subjected to 1.3 or 1.8 dS/m EC treated with Retrosal^®^ 0.2 mL/plant (Figures 4A,B). The photosynthetic water use efficiency (pWUE) did not show significant interaction between factors, while significant differences were found among biostimulant treatments in the 0.8 dS/m salinity (Figures 4C,D). The pWUE was higher in controls and decreased after biostimulant applications; a noticeable reduction occurred in Retrosal^®^ 0.2 mL/plant at 0.8 dS/m treated plants. Transpiration rate data (E) showed significant interaction (*p* < 0.05) between biostimulant and salinity (Figure 3B). Significant differences were also observed for biostimulant for *p* < 0.001. Since no significant interaction was found, data were also analyzed by one-way ANOVA. Results showed that E raised after biostimulant treatment at both doses; significant differences were observable in plants treated with Retrosal^®^ at 0.2 mL/plant compared to control plants under 1.3 or 1.8 dS/m EC.

#### Chlorophyll *a* Fluorescence

**FIGURE 5:**
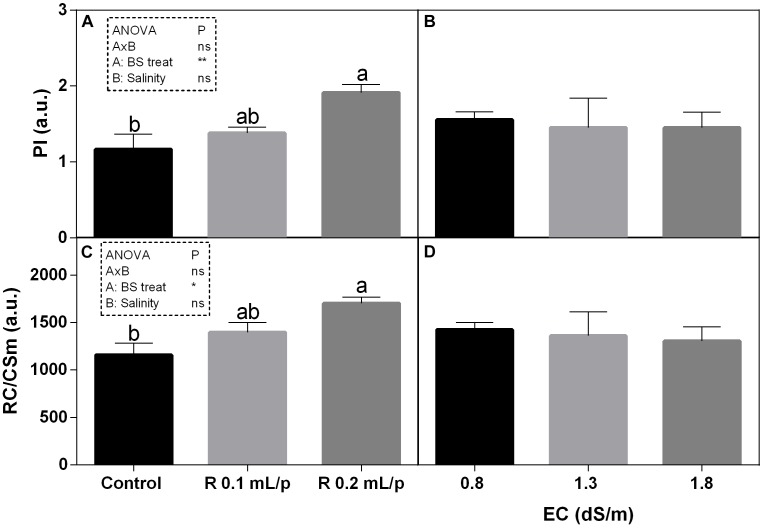
Chlorophyll *a* fluorescence parameters [performance index (PI) **(A,B)** and number of reaction centers per cross section **(C,D)**] measured *in vivo* in Romaine lettuce plants, at harvest. Plants were subjected to different levels of salinity (0.8, 1.3, and 1.8 dS/m) and treated with water (control) or Retrosal^®^ at 0.1 or 0.2 mL/plant dose. Data were compared by using two way ANOVA, with Tukey’s multiple comparison test (^∗^*p* < 0.05, ^∗∗^*p* < 0.01). Since there was not significant AxB interaction, the effect of BS treatments and EC levels was evaluated separately, comparing the respective means ± SE (*n* = 3). Different letters indicate statistical differences for *p* < 0.05.

Among the different JIP index, the performance index (PI) and the number of reaction centers per cross section (RC/CSm) have been reported and both showed the same pattern (Figure 5). Statistical analysis showed significant differences for the biostimulant factor, while salinity and interaction between the two factors were not significant. In fact, treatments with the biostimulant at 0.2 mL/plant dose induced a slight increase in these parameters than controls. Significant differences were observed in plants under 1.3 dS/m salinity level. PI ranged from 0.78 in control plants to 2.12 in the Retrosal^®^ 0.2 mL/plant subjected to 1.3 dS/m of salinity (Figures 5A,B). The RC/CSm were significantly different in plant at 1.3 dS/m EC, between control and the Retrosal^®^ 0.2 mL/plant treatment (Figures 5C,D).

**FIGURE 6:**
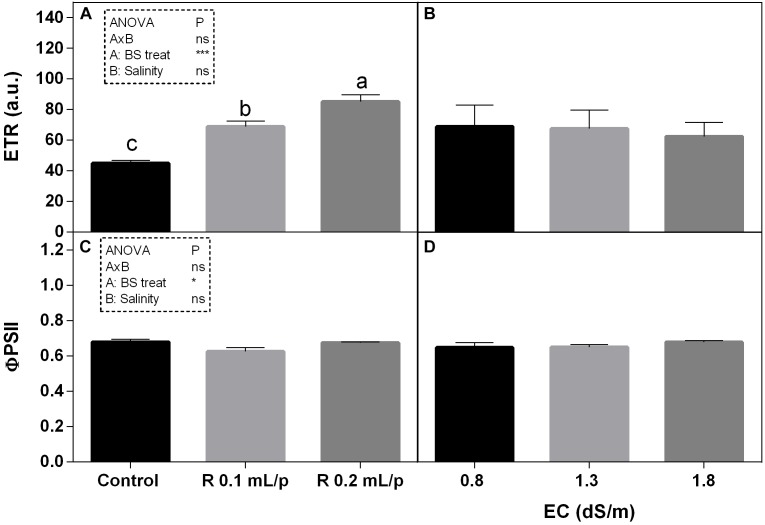
Modulated chlorophyll *a* fluorescence parameters [electron transport rate (ETR) **(A,B)** and photosystem II quantum efficiency **(C,D)**] measured *in vivo* in Romaine lettuce plants, at harvest. Plants were subjected to different levels of salinity (0.8, 1.3, and 1.8 dS/m) and treated with water (control) or Retrosal^®^ at 0.1 or 0.2 mL/plant dose. Data were compared by using two way ANOVA, with Tukey’s multiple comparison test (^∗^*p* < 0.05, ^∗∗∗^*p* < 0.001). Since there was not significant AxB interaction, the effect of BS treatments and EC levels was evaluated separately, comparing the respective means ± SE (*n* = 3). Different letters indicate statistical differences for *p* < 0.05.

As regards the modulated chlorophyll *a* fluorescence measurements, the electron transport rate (ETR) and the quantum efficiency of the photosystem II (ΦPS2) are reported (Figure 6). The two-way ANOVA for ETR and ΦPS2 showed significant values for the biostimulant factor, while the interaction and the salinity were not significant. ETR showed significant increases after biostimulant applications at all salinity levels (Figures 6A,B) in particular at 0.2 mL/plant dose. The ΦPS2 (Figures 6C,D) was significantly higher in 0.2 mL/plant dose treated leaves with 0.8 dS/m salinity level.

#### Total Chlorophylls, Carotenoids, and Sugars

**FIGURE 7:**
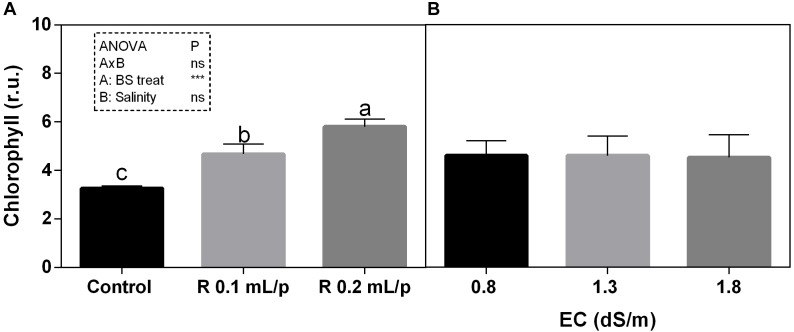
Chlorophyll content, measured *in vivo*, of Romaine lettuce leaves at harvest, subjected to different levels of salinity (0.8, 1.3, and 1.8 dS/m) and treated with water (control) or Retrosal^®^ at 0.1 or 0.2 mL/plant dose. Data were compared by using two way ANOVA, with Tukey’s multiple comparison test (^∗∗∗^*p* < 0.001). Since there was not significant AxB interaction, the effect of BS treatments **(A)** and EC levels **(B)** was evaluated separately, comparing the respective means ± SE (*n* = 3). Different letters indicate statistical differences for *p* < 0.05.

**FIGURE 8:**
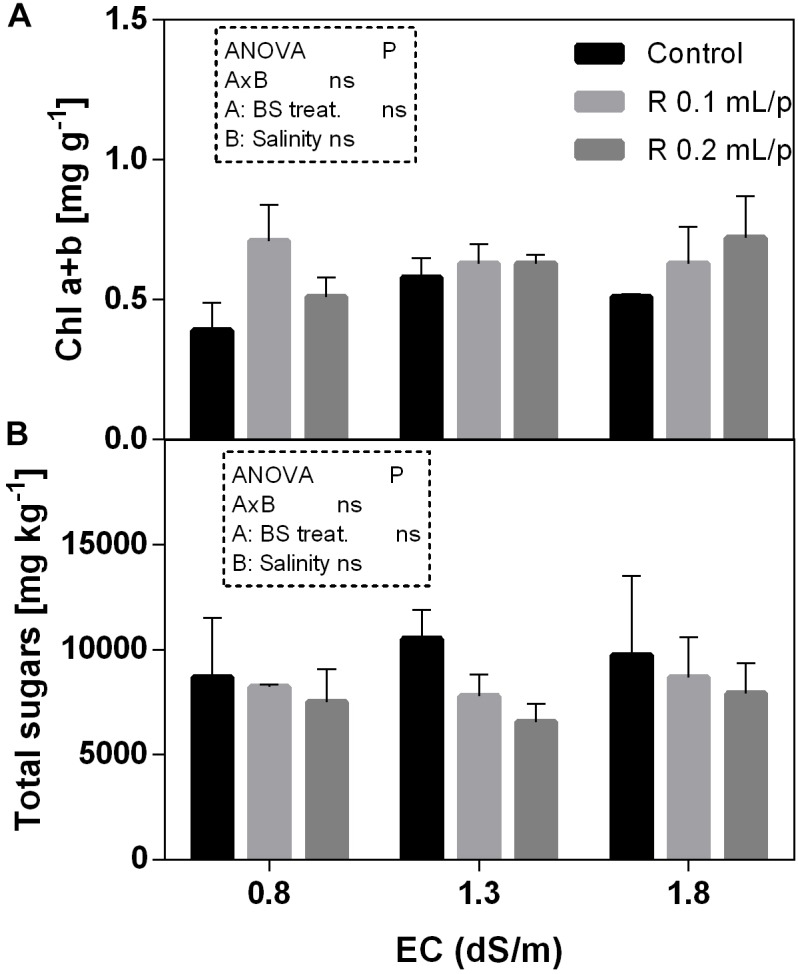
Chlorophyll *a+b* **(A)** and total sugars **(B)** concentrations in Romaine lettuce leaves, at harvest, subjected to different levels of salinity (0.8, 1.3, and 1.8 dS/m) and treated with water (control) or Retrosal^®^ at 0.1 or 0.2 mL/plant dose. Values are means ± SE (*n* = 3). Data were compared by using two way ANOVA, with Tukey’s multiple comparison test (*p* < 0.05).

**FIGURE 9:**
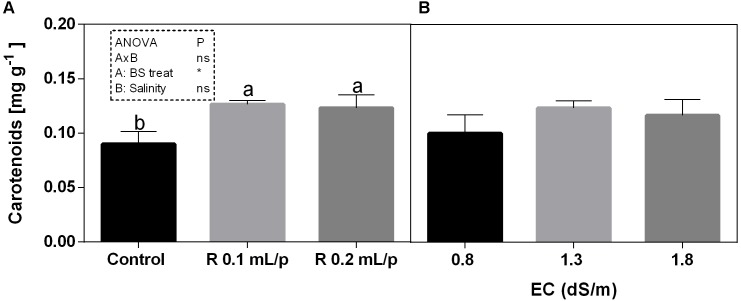
Carotenoids concentrations in Romaine lettuce leaves, at harvest, subjected to different levels of salinity (0.8, 1.3, and 1.8 dS/m) and treated with water (control) or Retrosal^®^ at 0.1 or 0.2 mL/plant dose. Data were compared by using two way ANOVA, with Tukey’s multiple comparison test (^∗^*p* < 0.05). Since there was not significant AxB interaction, the effect of BS treatments **(A)** and EC levels **(B)** was evaluated separately, comparing the respective means ± SE (*n* = 3). Different letters indicate statistical differences for *p* < 0.05.

The two-way ANOVA analysis showed that the interaction between factors was not significant, as well as the salinity, for all the determinations considered. On the contrary, the biostimulant factor was significant (*p* < 0.001) for the chlorophyll *in vivo* and carotenoids. It is possible to notice that the chlorophyll content measured *in vivo* in lettuce leaves (Figure 7) showed similar values in controls. Retrosal^®^ induced an increment of chlorophylls, confirmed by statistical analyses in leaves treated with the biostimulant 0.2 mL/plant at 1.3 or 1.8 dS/m salinity level. The destructive determinations showed the same pattern for chlorophylls *a+b* concentration and carotenoids (Figures 8A, 9). Total carotenoids showed values of 0.07 mg/g FW in controls and 0.14 mg/g FW in leaves of treated plants at 0.2 mL/plant dose. In fact, biostimulant treatment caused a slightly increment of the considered pigments, however the effect was not statistically relevant compared to controls.

The total sugars concentration in lettuce leaves did not show significant differences among treatments (Figure 8B).

#### Nitrate Levels and Proline

**FIGURE 10:**
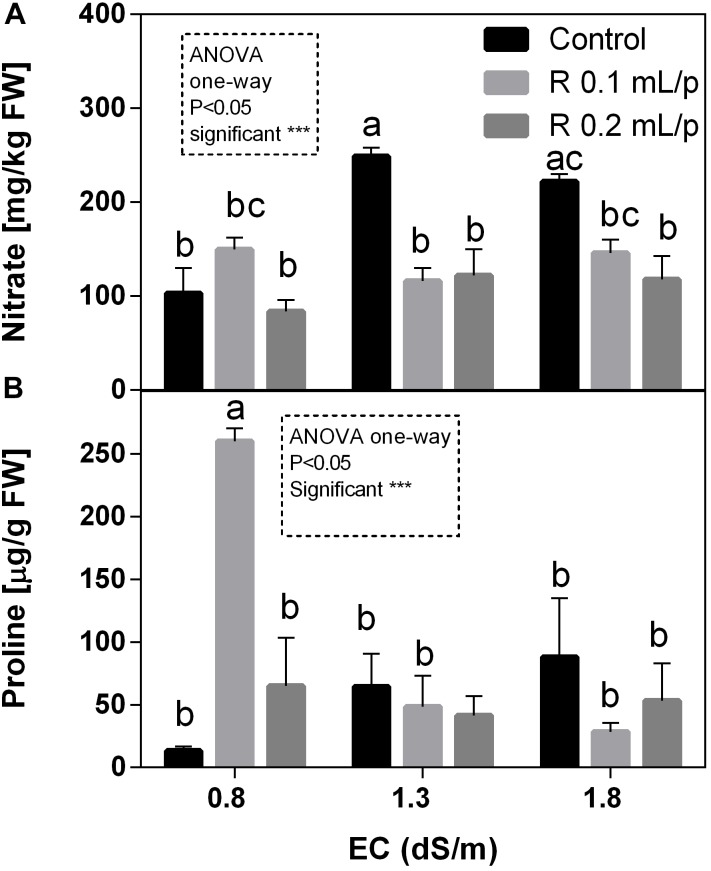
Nitrate **(A)** and proline **(B)** concentration in Romaine lettuce leaves, at harvest, subjected to different levels of salinity (0.8, 1.3, and 1.8 dS/m) and treated with water (control) or Retrosal^®^ at 0.1 or 0.2 mL/plant dose. Values are means ± SE (*n* = 3). Data were compared by using two way ANOVA, with Tukey’s multiple comparison test (^∗∗^*p* < 0.01, ^∗∗∗^*p* < 0.001). Since interaction was significant, data were also subjected to one-way ANOVA. Different letters indicate statistical differences for *p* < 0.05.

The two-way ANOVA analysis for nitrate data showed that interaction and factors were statistically significant. Therefore, data of all treatments were analyzed using one-way ANOVA and results indicated that biostimulant significantly reduced the nitrate concentration at 1.3 and 1.8 EC levels. In fact, treated plants showed values similar to the control 0.8 dS/m EC, suggesting that Retrosal^®^ allowed keeping lower nitrate levels. Nitrate values ranged from 83.7 to 248.7 mg kg^-1^ FW (Figure 10A). The graph shows that the increment of salinity caused a sensible increase of nitrate in leaves of control plants.

As observed for nitrate, the two-way ANOVA showed high significance (*p* < 0.001) for all factors and their interaction in the proline data (Figure 10B). In control plants it is possible to observe that the increasing levels of salinity caused a raise in proline concentration. Biostimulant treatment allowed maintaining proline levels lower, except in leaves treated with the biostimulant at 0.1 mL/plant dose at 0.8 dS/m EC, that showed the highest concentration observed. This result was also confirmed by one-way ANOVA analysis.

#### Abscisic Acid

**FIGURE 11:**
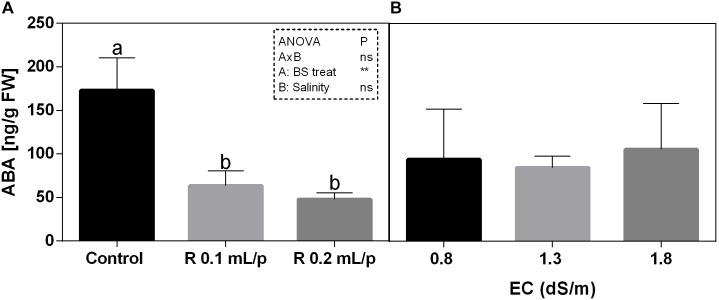
Abscisic acid (ABA) concentration in Romaine lettuce leaves, at harvest, subjected to different levels of salinity (0.8, 1.3, and 1.8 dS/m) and treated with water (control) or Retrosal^®^ at 0.1 or 0.2 mL/plant dose. Data were compared by using two way ANOVA, with Tukey’s multiple comparison test (^∗∗^*p* < 0.01). Since there was not significant AxB interaction, the effect of BS treatments **(A)** and EC levels **(B)** was evaluated separately, comparing the respective means ± SE (*n* = 3). Different letters indicate statistical differences for *p* < 0.05.

The statistical analysis revealed that the biostimulant factor was significant for *p* < 0.01, while the interaction and salinity were not significant (Figure 11). All plants treated with Retrosal^®^ showed lower values of ABA compared to control. A sensible decrease of ABA concentration was recorded after biostimulant application at 0.1 or 0.2 mL/plant dose at 0.8 and 1.8 dS/m EC.

#### Mineral Content

**FIGURE 12:**
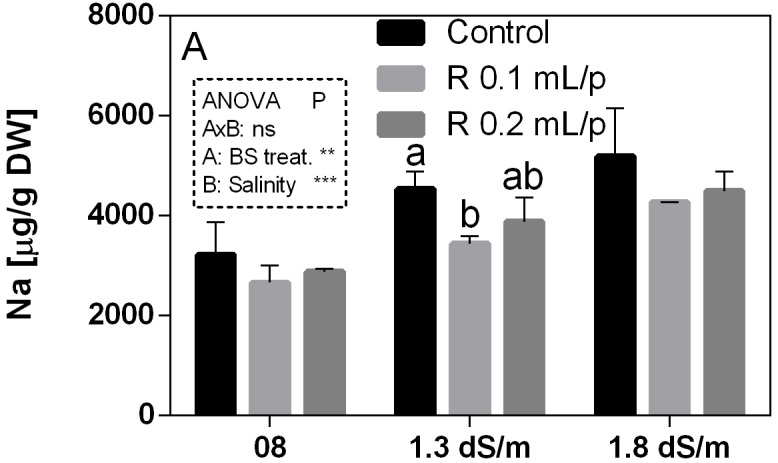
Sodium (Na) concentration in Romaine lettuce leaves, at harvest, subjected to different levels of salinity (0.8, 1.3, and 1.8 dS/m) and treated with water (control) or Retrosal^®^ at 0.1 or 0.2 mL/plant dose. Values are means ± SE (*n* = 3). Data were compared by using two way ANOVA, with Tukey’s multiple comparison test (^∗∗^*p* < 0.01, ^∗∗∗^*p* < 0.001). Different letters indicate statistical differences for *p* < 0.05.

The mineral content was determined in leaves at harvest. Mineral concentrations are reported in the Supplementary Table S1. Since the work was focused on salinity exposure, the values of sodium (Na) and calcium (Ca) have been discussed in relation to biostimulant applications. Na was strongly affected by the salinity (*p* < 0.0001) and increased with the increment of EC levels. The interaction was not significant, while the biostimulant factor was significant for *p* < 0.001. The lowering effect of the biostimulant in the Na leaf accumulation was evident in plants grown under 1.3 dS/m EC, treated with 0.1 mL/plant dose (Figure 12). The Ca content in leaves (Supplementary Table S1) did not show significant differences, even if a slightly increment was noticeable in leaves after the application of 0.2 mL/plant dose, at 1.8 dS/m EC level.

## Discussion

Plant responses to salinity differ greatly among species and to a lesser extent among varieties (Shannon and Grieve, 1998; Parida and Das, 2005). Their sensitivity is higher during seedling and reproductive stage (Machado and Serralheiro, 2017; Negrão et al., 2017). Furthermore, several environmental factors (temperature, wind, relative humidity, radiation, air pollution) show significant interaction with salinity effects (Shannon, 1997). Another environmental hazard that can be aggravated by salinity is the root zone waterlogging; in fact, root zone salinity, and waterlogging greatly increase the salt uptake compared with normal soil conditions (Shannon, 1997). The negative effect of salt stress can be commonly observed on growth reduction of plants (Shannon and Grieve, 1998; Santos and Caldeira, 1999; Akram et al., 2012). A decrease in fresh mass was observed in lettuce plants cv. Vera by Andriolo et al. (2005) and in a work of De Pascale and Barbieri (1995) conducted on lettuce, endive, and fennel. As described by Ünlükara et al. (2008), the yield of plants of lettuce cv. Crispa maintained steady values up to a threshold of salinity tolerance and then decreased with the increment of the soil salinity. The biostimulant tested in this work increased significantly the FW of lettuce plants compared to control. The enhancement in the growth of lettuce plants, after treatments, could be attributed to an increased nutrient uptake, as reported by Türkmen et al. (2004), who used humic acids in combination with Ca to treat tomato seedlings. In recent years, the functions of Ca were studied in particular for its role as a second messenger in the signal conduction between environmental factors and plant responses, in terms of growth and development (Kaya et al., 2002; Hepler, 2005). Free Ca is directly involved in the activation of salt overlay sensitive (SOS) sodium channels. The Ca acts as an inhibitor of sodium channels and reduces the uptake in cells. These findings are in agreement with our results observed in plants treated with Retrosal^®^ 0.2 mL/plant at the highest salinity. In fact, Na declined while Ca slightly increased, although not significant differences were observed.

Lucini et al. (2015) observed that applications of plant-derived protein hydrolysate mitigated the deleterious effects of salt stress (3.5 dS/m EC) on lettuce cv. Regina di Maggio. These results were consistent with a previous study of Ertani et al. (2013), who observed that a protein hydrolysate biostimulant derived from alfalfa increased maize plant biomass, even under salinity. The effect of biostimulants can be direct on the salt sensitivity but also indirect, increasing plant biomass and fastening the growing cycle. The application of Retrosal^®^ significantly increased the development rate, indicating a clear biostimulant effect.

Salt stress was demonstrated to affect negatively also the leaves photosynthetic pigment contents (Smirnoff, 1998; Türkmen et al., 2004). In fact, stress conditions led to an inhibition of chlorophyll biosynthesis, along with the activation of the pigments degradation by enzyme chlorophyllase (Santos, 2004). In the present work, results indicated that biostimulant treatments had a positive effect on the chlorophyll content measured *in vivo* compared to controls. Biostimulant applications preserved leaves pigments, contributing to maintain a good produce visual appearance and nutraceutical properties. Biostimulants are often able to increase leaf pigments concentrations; this concerns in particular products containing seaweed extracts, plant extracts, humic acid (Bulgari et al., 2015, 2017; Chbani et al., 2015; and references therein).

To evaluate the health-status of the photosynthetic apparatus in response to stress factors, the gas exchange analysis is a useful non-destructive method. Salinity has direct impact on the primary metabolism. The reduction of water uptake in salt stress conditions limits the photosynthesis. The excess of energy absorbed from the leaf must be dissipated to avoid leaf photo-damages. Among non-destructive methods, the chlorophyll *a* fluorescence and derived JIP indexes can be useful to monitor the progress of stress conditions, as well as leaf gas exchanges. These tools can be also used for evaluating the efficacy of biostimulant treatments (Bulgari et al., 2017). PI is an overall evaluation parameter of leaf functionality and it is associated to leaf health status. This index has been widely used for assessing plant performance under stress (Mehta et al., 2010) or to evaluate the effect of treatments (Misra et al., 2001; Cocetta et al., 2016). In our experiment, biostimulant treatment at 0.2 mL/plant dose induced a slight increase in PI than controls, and this increment was statistically significant at 1.3 dS/m EC level. Biostimulant also enhanced the ETR, indicating that higher electron flux was destined to the photosynthetic machinery. This higher energy use can be also explained by the higher number of active RC/CSm, which normally declined under salinity (Ferrante et al., 2011). Results suggested that, in our experimental conditions, a general positive effect deriving from the application of biostimulant was observable on net photosynthesis rate. Consistent results, regarding the effect of biostimulants on parameters of photosynthetic activity, were found, among others, in rocket treated with biostimulants of vegetable origin (Abdalla, 2013), in strawberry after seaweed extract application (Spinelli et al., 2010), in maize under drought treated with fulvic acid (Anjum et al., 2011), and also in ornamental plants after the application of a mix of plant extract rich in fulvic acids, humic acids, amino acids, and glycine betaine (Massa et al., 2016). To sum up, since photosynthesis has been measured at harvest it cannot be ruled out that it decreases at a later stage along with stomatal conductance. However, our data suggest that Retrosal^®^ could stimulate the crop performance by keeping open stomata, maintaining photosynthesis, source-sink relations (growth), and thus protecting from possible photoinhibition/photooxidation effects (Castro et al., 2012; Massa et al., 2016). Generally speaking, soluble sugars tend to increase in plants under salt stress, while starch content decreases (Chaves, 1991; Baki et al., 2000). However, as reported by Ashraf and Harris (2004), the role of carbohydrates in the salinity tolerance is not clear and further investigations are needed to conclude that they are universally associated with salt tolerance, because the variations in the accumulation of these compounds could vary among species. In our material, the tissue levels of total sugars were not affected by salinity and treatment applications, in fact all plants showed similar concentrations. These results indicated that treated plants did not show salinity stress under the conditions applied.

On the contrary, nitrate levels were affected by salinity; a sensible increase of nitrate was observable in control plants. Biostimulant treatments allowed maintaining nitrate concentration similar to the controls. The reduction of nitrate concentration in leaves is probably due to the increase of the nitrate assimilation by the activation of the nitrate and nitrite reductase enzymes. A reduction of nitrate after biostimulant application was observed in several species of leafy vegetables (Vernieri et al., 2005; Liu et al., 2007; Dudaš et al., 2016). The capability to keep nitrates low and under the limits imposed by EU regulations (Reg. No. 1258/2011) is very interesting in this commercial sector. The low nitrate concentrations observed are commonly found in Romaine lettuce as previously reported by Bulgari et al. (2017). The low nitrate accumulation also depends by the fertilizers application as well as from genotype, environmental, and management factors (Cometti et al., 2011). In our experiment, slow-release fertilizer was supplied at transplanting and during cultivation for satisfying the plant’s requirements. This strategy avoided the high accumulation of nitrate in leaves.

Nitrate and salinity are inversely correlated, because under stress conditions the sodium is accumulated in vacuoles avoiding the storage of nitrates that are straightly assimilated or not absorbed from the soil or nutrient solution. However, this behavior occurs when the plants are under severe salinity stress, while at beginning of the stress plants can also counteract the salinity by increasing the osmotic potential, by accumulating osmolytes, including nitrates. The initial increase of nitrate concentration under 10–20 mM NaCl salinity exposure was observed in Lettuce (*Lactuca sativa* L. subsp. capitata) grown in floating system, while declined at 30 mM (Tesi et al., 2003). Analogous results were also observed for lettuce grown in soilless cultivation, in which the nitrate concentration increased up to 2.5 dS/m and declined in the plants grown under 3.5 dS/m of salinity (Serio et al., 2001). Our results demonstrated that the nitrate content did not significantly change compared to the untreated and unstressed control after biostimulant applications, demonstrating the role of this product to alleviate the exposure to saline solutions.

Proline accumulates in many plant species under to a broad range of environmental stress conditions (Ashraf and Harris, 2004; Claussen, 2005; Rejeb et al., 2014; Xiong et al., 2014). Nowadays it is known that proline has multifunctional roles in plants (Szabados and Savouré, 2010). In addition, proline being an osmoprotectant, can act as a potent non-enzymatic antioxidant. In our treatments, we observed that the increasing levels of salinity caused a raise in proline concentration in control plants. Proline can play an important role in the osmotic adjustment and may participate to the scavenging of reactive oxygen species. Retrosal^®^ treatments, in general, allowed maintaining lower the proline levels under salinity. On the contrary, the highest concentration was found in leaves treated with the biostimulant at 0.1 mL/plant dose at 0.8 dS/m. These results prove a kind of dose depending effect of treatments on lettuce and support the hypothesized positive role of biostimulants in protecting plants from saline exposure. Further investigation should be performed to better understand the role of this biostimulant in the proline metabolism.

Abscisic acid is an essential phytohormone that regulates various aspects of plant growth and development in response to abiotic stress (Fujita et al., 2011). In stressful conditions, such as salinity, ABA content increases and it triggers the expression of many genes encoding various proteins important for biochemical and physiological processes (Xiong et al., 2014 and references therein). When lettuce plants were harvested at commercial maturity stage, all plants treated with biostimulant showed lower values of ABA compared to controls and in some cases this decrease was statistically significant. A reduction in ABA content in salt-stressed and biostimulant-treated plants might be related to the de-activation of ABA signaling pathways which controls the stomata closure (Trivellini et al., 2016). In fact, in our experiment the biostimulant treatments enhanced the stomatal conductance and this behavior might be reflected by a slightly improvement of net photosynthetic rate and an enhancement of FW. Thus, the treatment of plants with selected biostimulant agents during their development was accompanied by significant amelioration of stress impacts on plant physiology and growth. Moreover, similar findings were observed in a field study with pistachio (*Pistacia vera*), in which biostimulant treatments ameliorated negative effects on plant growth resulting from irrigation with low to moderate rates of NaCl. This effect was related to a reduction in proline accumulation and decreased levels of ABA in leaves of treated plants compared to controls (Moghaddam and Soleimani, 2012). Additionally, in plants grown under different stressful conditions, a decreased level of free ABA after application of biostimulant has been shown (Przybysz et al., 2010, 2014), suggesting again that changes in ABA concentration by lowering its accumulation resulted in a general positive effect on leaf gas exchanges and stimulated growth under salinity, as it was recorded also in this work.

## Conclusion

Crops are subjected to abiotic and artificial-induced stresses during their life span that greatly reduce productivity and also the quality of these commodities.

The preliminary results reported in this study indicate that the application of the biostimulant Retrosal^®^ on lettuce confers enhanced tolerance when plants are exposed to NaCl treatments, due to its multifaceted action at both biochemical and physiological level. In particular, we noted a significant biostimulant effect on several variables examined, among which fresh yield, dry biomass, chlorophyll content *in vivo*, nitrate concentration and some leaf gas exchange parameters as well as chlorophyll *a* fluorescence parameters. Thus, this biostimulant represents an effective tool to employ in crop management to stimulate plant growth and productivity. Further experiments will be necessary in order to investigate in depth the effects of Retrosal^®^ against salinity, subjecting lettuce plants to higher salinity concentrations, that could result more stressful for the considered crop.

## Author Contributions

RB performed the experiments and analytical determinations, and contributed to manuscript writing. AT contributed to ABA extraction and determination, and manuscript writing. AF was responsible for the research activities, experimental plan, and revision of the final manuscript.

## Conflict of Interest Statement

The authors declare that the research was conducted in the absence of any commercial or financial relationships that could be construed as a potential conflict of interest.

## Supplementary Material

FIGURE S1 Percentage dry matter of Romaine lettuce plants, at harvest. Plants were subjected to different levels of salinity (0.8, 1.3, and 1.8 dS/m) and treated with water (control) or Retrosal^®^ at 0.1 or 0.2 mL/plant dose. Values are means ± SE (*n* = 3). Data were compared by using two way ANOVA, with Tukey’s multiple comparison test.

TABLE S1 Concentration of mineral elements in lettuce leaves treated with water (control) or Retrosal^®^ 0.1 or 0.2 mL/plant. Data are means with standard deviations (*n* = 4).

TABLE S2 Electrical conductivity measured from substrate extract (1:2.5 v/v) where lettuce plants treated with water (control) or Retrosal^®^ 0.1 or 0.2 mL/plant were grown.
