# Supplementary figures and images for: Effects of Two Doses of Organic Extract-Based Biostimulant on Greenhouse Lettuce Grown Under Increasing NaCl Concentrations
Source: Front Plant Sci. 2019 Jan 7;9:1870. doi: 10.3389/fpls.2018.01870 (PMC6330896; doi:10.3389/fpls.2018.01870)

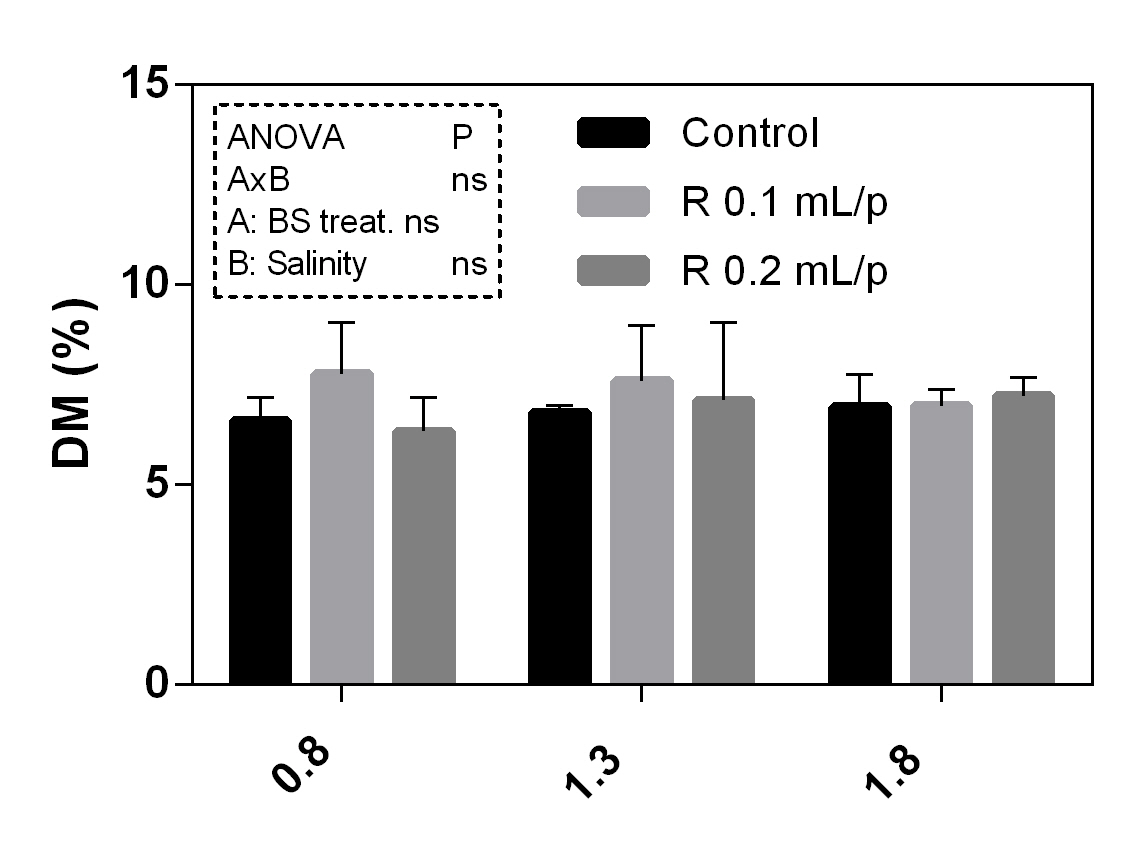

Supplement: FIGURE S1 — Percentage dry matter of Romaine lettuce plants, at harvest. Plants were subjected to different levels of salinity (0.8, 1.3, and 1.8 dS/m) and treated with water (control) or Retrosal® at 0.1 or 0.2 mL/plant dose. Values are means ± SE (n = 3). Data were compared by using two way ANOVA, with Tukey’s multiple comparison test. [file Image_1.JPEG]
