# Supplementary material for: Effects of Two Doses of Organic Extract-Based Biostimulant on Greenhouse Lettuce Grown Under Increasing NaCl Concentrations
Source: Front Plant Sci. 2019 Jan 7;9:1870. doi: 10.3389/fpls.2018.01870 (PMC6330896; doi:10.3389/fpls.2018.01870)
Supplement: TABLE S1 — Concentration of mineral elements in lettuce leaves treated with water (control) or Retrosal® 0.1 or 0.2 mL/plant. Data are means with standard deviations (n = 4). [file Table_1.DOCX]

Suppl. Table 1. Concentration of mineral elements in lettuce leaves treated with water (control) or Retrosal® 0.1 or 0.2 mL/plant. Data are means with standard deviations (n = 4).

|  | Salinity | **Mg** | **K** | **Ca** | **Mn** | **Fe** | **Cu** | **Zn** | **Cd** | **P** |
| --- | --- | --- | --- | --- | --- | --- | --- | --- | --- | --- |
|  | dS/m | **µg/g** | **µg/g** | **µg/g** | **µg/g** | **µg/g** | **µg/g** | **µg/g** | **µg/g** | **µg/g** |
| Control | 0.8 | 6155.7±591.08 | 98241.8±2994.58 | 10901.1±1934.30 | 139.36±15.59 | 198.45±7.10 | 9.69±1.02 | 118.16±8.27 | 0.49±0.30 | 12362.14±148.90 |
|  | 1.3 | 6059.961009.84 | 85344.6±16.42 | 10728.5±2554.30 | 155.05±31.38 | 212.11±24.01 | 11.84±0.84 | 123.30±13.84 | 0.31±0.08 | 12597.04±859.23 |
|  | 1.8 | 5968.60415.72 | 89611.7±0.42 | 10832.4±1024.91 | 128.97±3.33 | 189.12±24.24 | 11.73±2.58 | 113.87±1.04 | 0.35±0.10 | 12522.45±666.34 |
| Retrosal® 0.1 mL/p | 0.8 | 5543.9±547.81 | 91791.2±1325.63 | 10531.8±542.48 | 69.19±1.99 | 211.25±32.29 | 8.48±0.14 | 112.16±8.38 | 0.28±0.04 | 10592.71±844.07 |
|  | 1.3 | 5477.9±494.13 | 91000.0±1333.22 | 10753.1±569.43 | 61.39±8.42 | 209.45±74.03 | 9.24±0.94 | 102.24±9.06 | 0.26±0.01 | 11123.65±333.30 |
|  | 1.8 | 5176.8±84.24 | 87373.6±5.13 | 9858.7±59.27 | 92.36±8.74 | 182.81±28.21 | 7.73±1.64 | 93.69±10.19 | 0.24±0.01 | 10307.25±154.91 |
| Retrosal® 0.2 mL/p | 0.8 | 5673.3±73.16 | 86112.2±0.29 | 11028.3±224.97 | 115.73±1.19 | 208.77±16.34 | 6.49±0.76 | 123.38±6.04 | 0.27±0.00 | 10672.79±262.69 |
|  | 1.3 | 5314.4±288.93 | 476622.3±551540.08 | 10604.3±489.73 | 108.61±3.90 | 186.93±12.91 | 8.51±0.74 | 117.60±8.36 | 0.34±0.01 | 10593.65±515.39 |
|  | 1.8 | 5883.3±83.33 | 92000.00±1334.10 | 12063.2±342.62 | 138.97±10.17 | 197.13±10.35 | 8.41±3.25 | 133.41±13.06 | 0.27±0.01 | 10645.41±684.99 |

Suppl. Table 2. Electrical conductivity measured from substrate extract (1:2.5 v/v) where lettuce plants treated with water (control) or Retrosal® 0.1 or 0.2 mL/plant were grown.

|  | Salinity | EC |
| --- | --- | --- |
|  | dS/m | dS/m |
| Control | 0.8 | 0.836 |
|  | 1.3 | 0.873 |
|  | 1.8 | 0.942 |
| Retrosal® 0.1 mL/p | 0.8 | 0.701 |
|  | 1.3 | 0.598 |
|  | 1.8 | 0.635 |
| Retrosal® 0.2 mL/p | 0.8 | 0.721 |
|  | 1.3 | 0.781 |
|  | 1.8 | 1.054 |
